# Stomatal responses to VPD utilize guard cell intracellular signaling components

**DOI:** 10.3389/fpls.2024.1351612

**Published:** 2024-02-05

**Authors:** Yotam Zait, Ariel Joseph, Sarah M. Assmann

**Affiliations:** ^1^ Biology Department, Penn State University, Mueller Laboratory, University Park, PA, United States; ^2^ The Robert H. Smith Institute of Plant Sciences and Genetics in Agriculture, Faculty of Agriculture, Food, and Environment, The Hebrew University of Jerusalem, Rehovot, Israel

**Keywords:** *Arabidopsis thaliana*, *Brassica napus*, *Oryza sativa*, heterotrimeric G proteins, guard cell, stomatal conductance, vapor pressure difference (VPD)

## Abstract

Stomatal pores, vital for CO_2_ uptake and water loss regulation in plants, are formed by two specialized guard cells. Despite their importance, there is limited understanding of how guard cells sense and respond to changes in vapor pressure difference (VPD). This study leverages a selection of CO_2_ hyposensitive and abscisic acid (ABA) signaling mutants in Arabidopsis, including heterotrimeric G protein mutants and RLK (receptor-like kinase) mutants, along with a variety of canola cultivars to delve into the intracellular signaling mechanisms prompting stomatal closure in response to high VPD. Stomatal conductance response to step changes in VPD was measured using the LI-6800F gas exchange system. Our findings highlight that stomatal responses to VPD utilize intracellular signaling components. VPD hyposensitivity was particularly evident in mutants of the *ht1* (*HIGH LEAF TEMPERATURE1*) gene, which encodes a protein kinase expressed mainly in guard cells, and in *gpa1-3*, a null mutant of the sole canonical heterotrimeric Gα subunit, previously implicated in stomatal signaling. Consequently, this research identifies a nexus in the intricate relationships between guard cell signal perception, stomatal conductance, environmental humidity, and CO_2_ levels.

## Introduction

Stomata, microscopic pores found in the leaf epidermis, play a pivotal role in controlling leaf CO_2_ uptake and transpirational water loss. Stomata are formed by pairs of specialized epidermal cells known as guard cells (Darwin and Pertz, 1911; Raschke, 1975). Guard cells actively regulate intracellular solute concentrations, which drive trans-membrane water flux and thus determine cell volume and turgor pressure (Jones and Mansfield, 1970; Assmann and Jegla, 2016). When the volume and turgor of guard cells increase, they bow apart, enlarging the pore aperture and thus promoting water loss from the leaf to the atmosphere. The driving force for this evaporation is the leaf-to-air vapor pressure difference (VPD), defined as the difference in water vapor partial pressure between the leaf’s substomatal cavity and the surrounding air. While numerous studies have sought to understand stomatal functioning, the mechanism explaining how stomata sense VPD and what triggers consequent changes in guard cell volume and stomatal apertures remains elusive, especially since VPD, relative humidity (RH), and transpiration are all interlinked. In particular, the mechanism underlying stomatal closure following short-term exposure to high VPD, believed to stem from metabolically-controlled solute release from guard cells (Grantz and Zeiger, 1986; Buckley, 2005), lacks substantial experimental evidence. Early observations that high VPD accelerates the rate of transient stomatal conductance changes in response to pulses of blue light in both the dicot, soybean, and the graminaceous monocot, sugarcane (Assmann and Grantz, 1990a; Assmann and Grantz, 1990b), are consistent with the hypothesis that VPD modulates intracellular signaling cascades, such as those well-documented (Shimazaki et al., 2007; Assmann and Jegla, 2016; Matthews et al., 2019) for the phototropin-mediated (Kinoshita et al., 2001; Hosotani et al., 2021) rapid blue light response.

Previous work has suggested possible involvement of localized “water loss sensors” in the guard cells, responding more to transpiration than to RH itself (Mott and Parkhurst, 1991). However, as VPD increases, transpiration can actually decline because of decreased stomatal conductance, obviating a monotonic relationship between stomatal conductance and transpiration that would be expected if transpiration were the parameter being sensed (Assmann and Grantz, 1990a; Assmann and Grantz, 1990b). Due to its important role in stomatal regulation, ABA has been considered as a possible key player in the process of stomatal closure after VPD perturbation (McAdam and Brodribb, 2016). Some work suggests that stomatal closure is driven by the rapid up-regulation of foliar ABA levels following a VPD-induced loss of leaf turgor (Bauer et al., 2013; McAdam and Brodribb, 2015). For example, in the dicot herb, *Senecio minimus*, small increases in VPD had no effect on stomatal conductance, while step increases in VPD calculated to be large enough to reduce leaf turgor both increased leaf ABA content and triggered decreases in stomatal conductance (Binstock et al., 2023). However, the observations that stomata in isolated epidermis show a VPD response (Lösch, 1977), and that ABA-deficient mutants exhibit a reduction in a stomatal aperture in response to VPD, just like wild type (Assmann et al., 2000; Merilo et al., 2018), raises a debate regarding the essentiality of ABA in the VPD response. In this study, we explore the possibility of shared intracellular signaling components between stomatal responses to VPD, elevated CO_2,_ ABA, and pathogens, in particular leveraging key CO_2_ hyposensitive mutants and mutants in signaling elements downstream of ABA in our experimental design (**Table 1**). In our study, while Arabidopsis serves as our reference plant, we also examined the VPD response of different cultivars of canola (*Brassica napus*), which belongs to the same plant family as Arabidopsis, and rice, representing monocot grasses. This broader investigation enables us to gain insights into stomatal responses across diverse plant species, enhancing our understanding of how different crops may adapt to VPD fluctuations in changing environmental conditions.

**Table 1:** Summary of the Arabidopsis mutants used in this study and the initial reference reporting a role for the gene in Arabidopsis guard cells.

Genotype	Gene	Category	Guard Cell Phenotype	Reference for Guard Cell Phenotype
**Col-0**	Wild type	Wild type	Wild type	
***ht1-1***	*HIGH LEAF TEMPERATURE1*	CO_2_ signaling kinase	Impaired in sensing or responding to CO_2_	Hashimoto et al., 2006
***ht1-2***	*HIGH LEAF TEMPERATURE1*	CO_2_ signaling kinase	Impaired in sensing or responding to CO_2_	Hashimoto et al., 2006
***slac1***	*SLOW ANION CHANNEL-ASSOCIATED1*	ABA/CO_2_ signaling anion channel	Impaired in sensing or responding to ABA/CO_2_	Vahisalu et al., 2008
***ost1***	*OPEN STOMATA1*	ABA signaling kinase	Impaired in sensing or responding to ABA	Yoshida et al., 2002
***ca1ca4***	*B CARBONIC ANHYDRASE1/4*	CO_2_ signaling carbonic anhydrases	Impaired in sensing or responding to CO_2_	Hu et al., 2010
***rhc1***	*RESISTANT TO HIGH CO_2_1*	CO_2_ signaling MATE transporter	Impaired in sensing or responding to CO_2_	Tian et al., 2015
***aha1-6***	*ARABIDOPSIS H^+^ ATPase1*	Plasma membrane H^+^-ATPase	Impaired in responding to blue and red light	Ando and Kinoshita, 2018
***gpa1-3***	*G PROTEIN ALPHA SUBUNIT1*	Heterotrimeric G protein subunit	Impaired in sensing or responding to ABA	Wang et al., 2001
***agb1-2***	*ARABIDOPSIS GTP BINDING PROTEIN BETA 1*	Heterotrimeric G protein β subunit	Impaired in sensing or responding to ABA	Fan et al., 2008
***Gα quad***	*G PROTEIN ALPHA SUBUNIT1/EXTRA-LARGE G-PROTEIN 1/2/3*	Heterotrimeric G protein *α* subunits	NA*	NA
***bak1-4***	*BRI1-ASSOCIATED RECEPTOR KINASE1*	Receptor-Like Kinases	Impaired in sensing or responding to ABA	Shang et al., 2016
***fls2***	*FLAGELLIN-SENSITIVE2*	Receptor-Like Kinases	Impaired in sensing or responding to flg22 elicitor	Zhang et al., 2008
***pskr1-pskr2***	*PHYTOSULFOKIN RECEPTOR1/2*	Receptor-Like Kinases	NA*	NA
***gcr1-2***	*G-COUPLED RECEPTOR1*	putative G protein coupled receptor	Altered in sensing or responding to ABA	Pandey and Assmann, 2004

*Not available.

The initial reference describing each mutant line is provided in Materials and Methods.

## Materials and methods

### Plant materials and growth conditions

#### Arabidopsis

The plant materials used in the study included *Arabidopsis thaliana* accession Col-0 and various T-DNA mutants in the Col-0 background (**Table 1**): *ca1ca4* (Hu et al., 2010)*, ht1-1, ht1-2* (Hashimoto et al., 2006)*, rhc1* (SALK_123674) (Tian et al., 2015)*, aha1-6* (SALK_016325) (Haruta et al., 2010), *ost1-3* (SALK_008068) (Yoshida et al., 2002), *slac1-3* (SALK_099139) (Vahisalu et al., 2008), *gpa1-3* (Gα mutant) (Jones et al., 2003), *xlg1-1xlg2-1xlg3-4gpa1-3* (Gα quadruple mutant), (Yu et al., 2018) which contains a knockdown allele of the *XLG1* gene (Ding et al., 2008) and knockout alleles of the other 3 genes, *agb1-2* (Gβ mutant) (Ullah et al., 2003), *bak1-4 (*SALK*_116202)* (Heese et al., 2007), *fls2 (SAIL_691_C04)* (Melotto et al., 2006), *gcr1-2* (Chen et al., 2006), *pskr1-3pskr2-2* (Hartmann et al., 2013). Sterilized seeds of these genotypes were placed on agarose medium containing 1% sucrose and half-strength Murashige and Skoog medium (MS), with a pH of 5.6. The seeds were kept on plates in the dark at 4°C for 96 hours for germination, and then the plates were placed vertically for 14 days under light (200 µmol photons m^−2^ s^-1^). Afterward, the seedlings were transplanted into 0.5L pots filled with Metro Mix 360 soil mounded into a cone shape to maximize leaf area and facilitate gas exchange measurements. The pots were allowed to exceed their normal soil capacity, thus enabling the rosette to grow downwards, which facilitated manipulations for gas exchange measurements. The plants were grown in a growth chamber with an 8-hour light/16-hour dark regime, under a light intensity of 200 µmol photons m^−2^ s^−1^. The temperature was set to 21/19°C (light/dark), and the relative humidity was maintained at approximately 60%. The plants were watered twice a week with deionized water to ensure the pots remained at full soil water capacity.

#### Rice

Both Taichung 65 (T65; WT) and *d1*, a protein null mutant of *RGA1*, which encodes the rice heterotrimeric Gα subunit (Oki et al., 2009), were grown in a greenhouse in 10L pots filled with a soil mixture (Green 20, Even Ari, Israel). The greenhouse maintained an average temperature of 30°C during the day and 22°C during the night.

#### Canola

Seeds of the following cultivars: AR91004, AR91907, Aspen, Bridger, Cascade, Cathy, CEI3, Erica, Flint, Hummus, IMC129, LB2135, Polo canola, Printol, Reston, Selkirk, Span, Tobin, Webster, and Wichita obtained from the USDA canola germplasm collection were sown in 6-inch pots filled with Metro-mix 360 potting mixture. The plants were grown in a growth chamber with an 8-hour light/16-hour dark regime, under a light intensity of 300 µmol photons m^−2^ s^−1^. The temperature was set to 21/19°C (light/dark), and the relative humidity was maintained at approximately 60 percent.

### Gas exchange measurements

**Figure 1:**
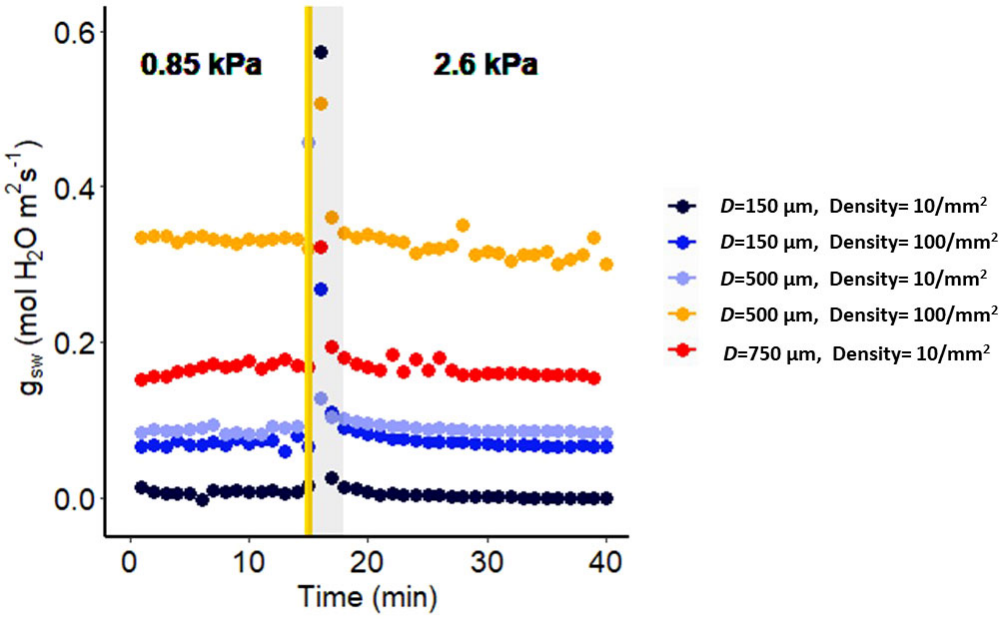
Effect of a sharp increase in vapor pressure difference (VPD) on conductance to water vapor of artificial plastic leaves with different pore diameters and densities. The vertical line in the graph indicates the time at which the VPD was changed from 0.85 to 2.6 kPa. The shaded region indicates data points that are not reliable.

Gas exchange measurements were conducted using the LI-6800F portable photosynthesis and fluorescence system (LI-COR Biosciences, Lincoln, NE, USA). The measuring chamber enclosed a circular 2-cm^2^ leaf area, such that the combined effect of gas fluxes from both sides of the leaf was assessed. The air-flow rate was kept constant at 300 µmol s^-1^, and the reference CO_2_ concentration was maintained at 400 µmol CO_2_ mol^-1^ air (ppm) with the leaf fan set to 5000 rpm. Prior to each measurement set, light intensity was adjusted to 400 µmol m^-2^ s^-1^ for Arabidopsis, 800 µmol m^-2^ s^-1^ for canola, and 1000 µmol m^-2^ s^-1^ for rice, using the LI-6800, with 10% blue light. Gas exchange measurements were conducted during the day (09:00 a.m. -15:00 p.m.) to avoid circadian influences on stomatal conductance. We selected young fully expanded leaves in Arabidopsis and canola, while the flag leaf was chosen in rice. To investigate the effects of vapor pressure difference (VPD) on stomatal conductance, VPD levels were adjusted for Arabidopsis using the LI-6800F humidifier, while maintaining a constant air temperature of 22°C. The relative humidity (RH) in the leaf chamber was first set to 70% (VPD of 0.65 kPa), and stomatal conductance was allowed to reach steady state, which required 20-30 minutes. Then, the humidity was rapidly reduced to 15% (VPD of 2.2 kPa). Measurements were recorded every minute for 40-60 minutes. To ensure rapid imposition of the RH change, the LI-6800 desiccators were oven-dried at 70°C overnight before each day of measurements. The level of stomatal closure was determined as the difference between the stomatal conductance at time zero and the stomatal conductance after reaching a new steady state. The “wrong way response” (WWR) was defined as a rapid increase in g_sw_ after the VPD change. The amplitude of the WWR was measured as the difference between the peak stomatal conductance after the humidity drop and the stomatal conductance at time zero. The criteria for assessing the duration of the WWR were calculated differently depending on the genotype’s behavior: For genotypes that experienced a reduction in stomatal conductance beyond that of the original baseline value, the duration was calculated as the time required for stomatal conductance to return to the same baseline value as observed before the humidity change, i.e., at time zero (Buckley et al., 2011). For genotypes where the new stable level of gsw after the step change was greater than that of the original baseline, the duration of the WWR was calculated from time zero to the point when they achieved this new steady-state value. This measurement thus captures the time required for stomatal conductance to reach and stabilize at a new steady-state. The cumulative water loss during the WWR was calculated as the summation of transpiration data throughout the duration of the WWR. Since we found that measurements in the first 3 minutes are not reliable (**Figure 1**), cumulative transpiration was calculated starting after 3 minutes from the humidity change. The VPD-induced change in g_sw_ was calculated as the difference between the steady-state g_sw_ values before and after the VPD step. For each set of experiments, we used different Col-0 plants to ensure the validity of our comparisons with the various mutants. In all, 15 different Arabidopsis genotypes and a total of 68 Arabidopsis plants were assayed in these experiments.

### LI-6800 measurements on artificial leaves

To validate that the stomatal conductance response to VPD measured by the LI-6800 instrument is not influenced by instrument artifacts, we conducted experiments using artificial leaves. These artificial leaves were carefully designed with known specifications, including a constant stomatal pore diameter and pore density, ensuring that they exhibit a constant stomatal conductance. The leaves were printed from Vero using a Stratasys J826™ Prime 3D printer. We positioned plastic leaves with a non-conductive, water-resistant surface, consisting of no pores on the adaxial side, and a conductive surface, consisting of pores of different diameters (150, 500, or 750 µm) and at different densities (1, 10, or 100 pores mm^-2^) on the abaxial side. We inserted filter paper (Whatman No. 3) saturated with distilled water within each leaf, ensuring a continuous water supply. The VPD levels within the leaf chamber were meticulously controlled using the LI-6800F humidifier, while maintaining air temperature of 25°C throughout the experiment. The CAD files for construction of artificial leaves are freely available at https://github.com/AssmannLab/Stomatal-responses-to-VPD-utilize-guard-cell-intracellular-signaling-components.

### Response to VPD in canola varieties

Six-week-old canola plants of the specified cultivars were enclosed within plastic transparent domes for 3 hours to create a controlled environment with a temperature of 22°C and a relative humidity of 80%, resulting in low VPD (0.52 kPa). During this time, eight measurements were obtained for each plant using a LI-600 leaf porometer/fluorometer (LI-COR Biosciences, Lincoln, NE, USA). To alter the VPD conditions, dry air was introduced by a heating fan, elevating the temperature to 28°C and reducing the relative humidity to 35% for 2 hours (VPD 2.45 kPa) prior to repeating measurements with the porometer. This manipulation of conditions created a high VPD environment for assessment of the response of stomatal conductance to VPD.

### Statistical analysis

To assess the differences in gas exchange parameters between the Col-0 (WT) and the studied mutant, we conducted two-sample t-tests using the base R statistical software [version 4.3.22 (https://cran.r-project.org)]. P values of comparisons are provided in **Supplementary Table 1**.

## Results

### Assessing the reliability of the LI-6800 instrument in measuring stomatal conductance response to VPD: artificial leaf experiments

Measuring humidity responses with a gas exchange system can be prone to artifacts due to a discernible delay between setting the desired relative humidity (RH) of the chamber and its actual attainment. This delay arises because the chamber has a finite volume, necessitating time for it to stabilize at the new environmental conditions. This delay can lead to instability in measurements, particularly affecting the precision of infrared gas analyzer (IRGA) readings, especially when abrupt changes occur in the input parameters to the chamber. Such single step changes in RH have the potential to significantly disrupt the accurate calculation of stomatal conductance. To assess the reliability of data collected using the LI-6800, we utilized artificial leaves as a means of evaluating the system’s readouts. Our study demonstrated that, for all constant conductance scenarios (pore size and density), the initial 3-minute period following a VPD change was particularly vulnerable to artifacts (**Figure 1**). In all artificial leaves the values of the leaf conductance returned after 3 minutes to the same level as recorded prior to the humidity change. This underscores the importance of caution in interpreting results and highlights the potential for erroneous responses when utilizing the LI-6800 system. Accordingly, in the gas exchange figures in this manuscript, we do not show or incorporate in calculations any datapoints acquired at t < 3 min after the step change in VPD.

### Exploring stomatal response to VPD in Arabidopsis and rice: mutant analyses and insights into sensitivity and signaling pathways

**Figure 2:**
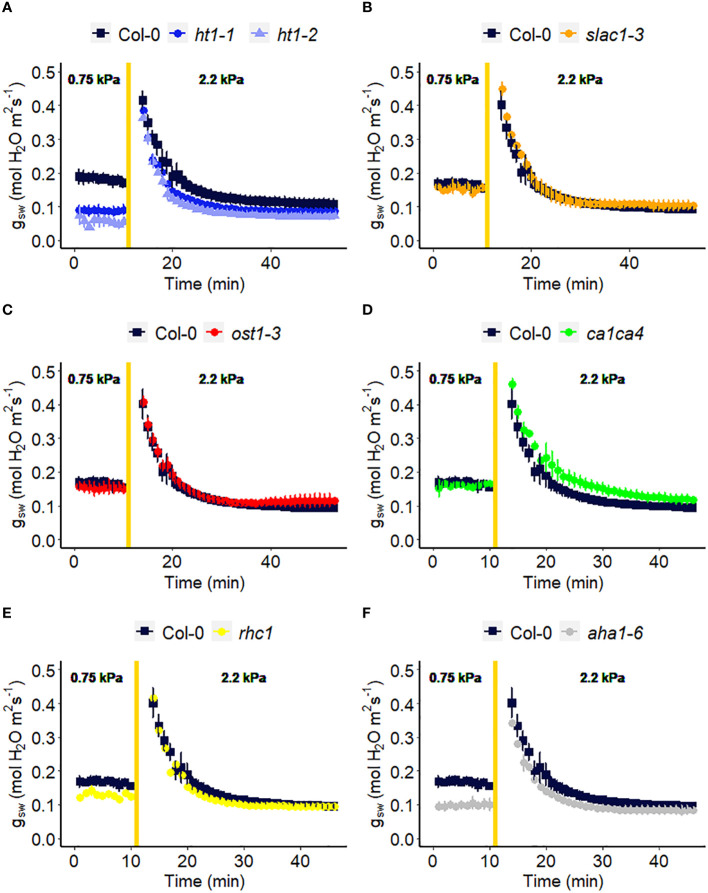
Effect of a sharp increase in vapor pressure difference (VPD) on stomatal conductance in Arabidopsis WT (Col-0) and the mutants: **(A)** *ht1-1, ht1-2*, **(B)** *slac1-3*, **(C)** *ost1-3*, **(D)** *ca1ca4*, **(E)** *rhc1*, and **(F)** *aha1-6*. The vertical line in the graph indicates the time at which the VPD was changed from 0.75 to 2.2 kPa. Error bars represent standard error of the mean (n=3). For ease of comparison, the same Col-0 data are plotted in each of the 6 panels.

**Figure 3:**
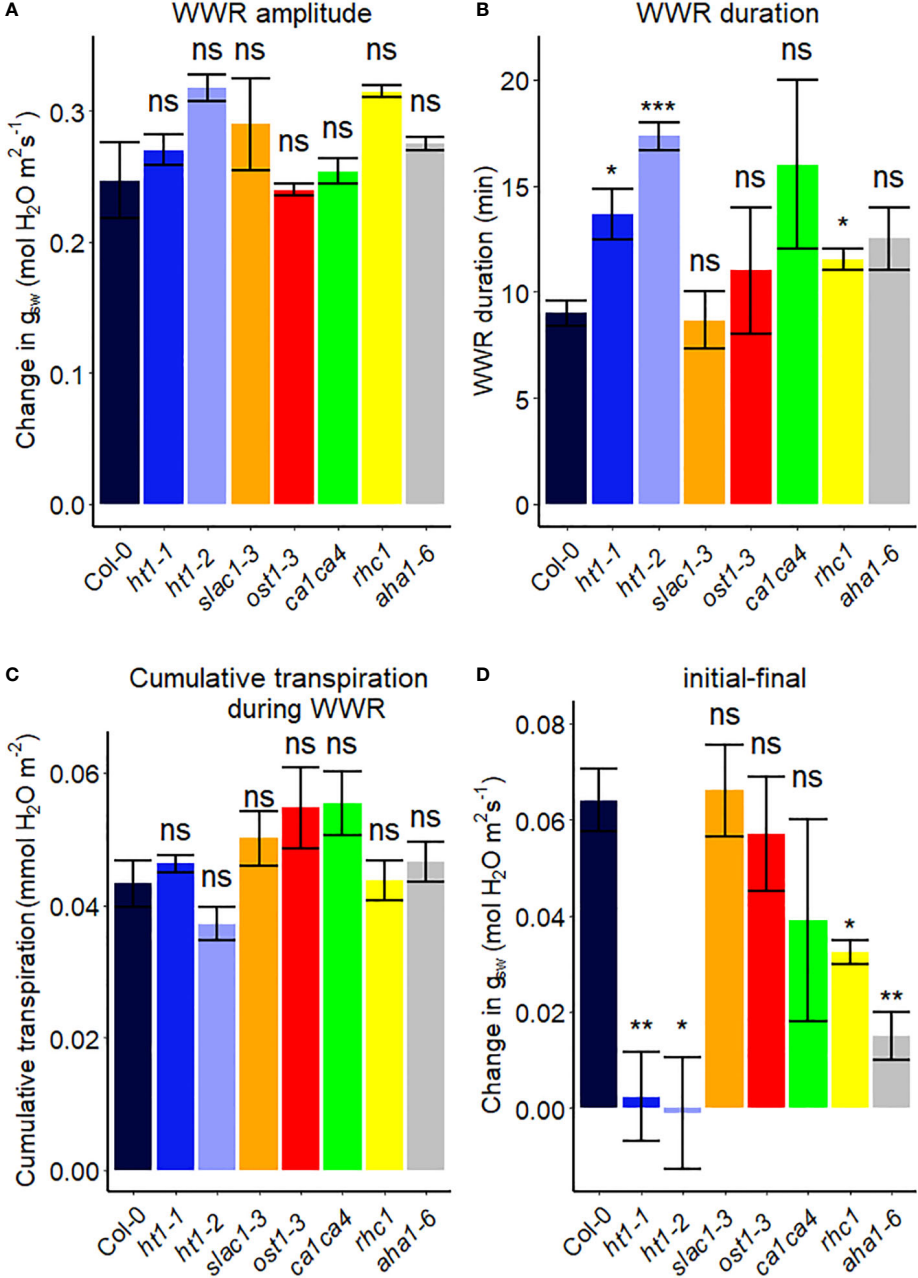
Stomatal VPD response characteristics in Arabidopsis WT (Col-0) and mutant plants (*ht1-1, ht1-2, slac1-3, ost1-3, ca1ca4, rhc1*, and *aha1-6*). **(A)** The “wrong way response” (WWR) amplitude represents the differences in stomatal conductance at the time of the peak vs. before the VPD change. **(B)** The WWR duration represents the duration between the steady-state conductance before the VPD change and returning to the same baseline. **(C)** Cumulative water loss in transpiration during WWR. **(D)** Difference in steady-state g_sw_ values before and after the change in VPD. Asterisks indicate p-values and define significance levels as follows: * for p < 0.05; ** for p < 0.01; *** for p < 0.0001; ns, not significant.

We assessed the response to a step increase in VPD of Arabidopsis wild-type (Col-0) vs. mutants known to be impaired in sensing or responding to CO_2_ (*ht1-1*, *ht1-2*, *slac1-3*, *ca1ca4*, *rhc1*) (Hashimoto et al., 2006; Vahisalu et al., 2008; Hu et al., 2010; Tian et al., 2015; Zhang et al., 2018), ABA (*ost1-3, slac1-3*) (Ache et al., 2010; Acharya et al., 2013) and light (*aha1-6*), (Yamauchi et al., 2016; Ando and Kinoshita, 2018). The gas exchange data (**Figure 2**) illustrate the rapid transient opening movement called the stomatal “wrong way response” (WWR), in which a decrease in epidermal cell turgor pressure will result in the guard cells transiently sinking into epidermal cells and increasing pore aperture followed by an oppositely directed closing movement resulting in a new steady state conductance. There were no significant differences in the WWR amplitude between genotypes (**Figure 3A**) but the duration of the WWR was on average 5 and 8 minutes longer in the *ht1-1* (P<0.05) and the *ht1-2* (P<0.001) mutants compared to Col-0 respectively (**Figures 3A, B**). The *rhc1* mutant also exhibited a broadened wrong way response (13 min) compared to Col-0 (9 min) (P<0.05; **Figure 3B**). No significant differences were observed among these genotypes in the cumulative water loss via transpiration during the WWR (**Figure 3C**). However, the steady-state response of stomatal conductance (*g_sw_*) to an increase in VPD from 0.75 kPa to 2.2 kPa in Col-0 vs. the mutants showed clear genotypic differences. Col-0 showed clear sensitivity to an increase of VPD from 0.75 kPa to 2.2 kPa VPD, seen as a stomatal conductance decrease below the initial baseline (**Figure 3D**). VPD sensitivity similar to that of the Col-0 wild-type was observed for the mutants *slac1-3*, *ost1-3*, and *ca1ca4.* However, there was essentially no VPD-induced change in steady-state conductance for the Arabidopsis mutants *ht1-1* (P<0.01), and *ht1-2* (P<0.05), while *rhc1* (P<0.05) and *aha1-6* (P<0.01) also exhibited hyposensitivity to VPD (**Figures 2**, **3**).

### Role of heterotrimeric G proteins in Arabidopsis and rice in the stomatal response to VPD

**Figure 4:**
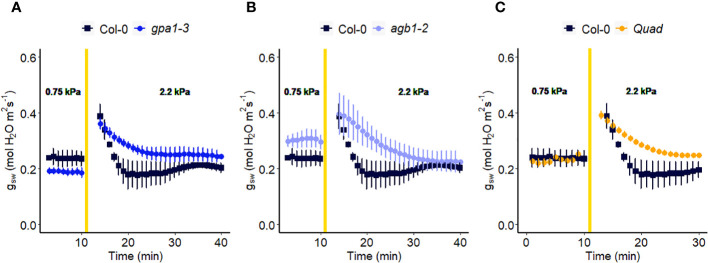
Effect of a sharp increase in vapor pressure difference (VPD) on stomatal conductance in Arabidopsis WT (Col-0) and the mutants: **(A)** *gpa1-3*, **(B)** *agb1-2*, and **(C)** Gα quadruple mutant *xlg1-1xlg2-1xlg3-4gpa1-3*. The vertical line in the graph indicates the time at which the VPD was changed from 0.75 to 2.2 kPa. Error bars represent standard error of the mean (n=3-5). For ease of comparison, the same Col-0 data are plotted in each of the 3 panels.

**Figure 5:**
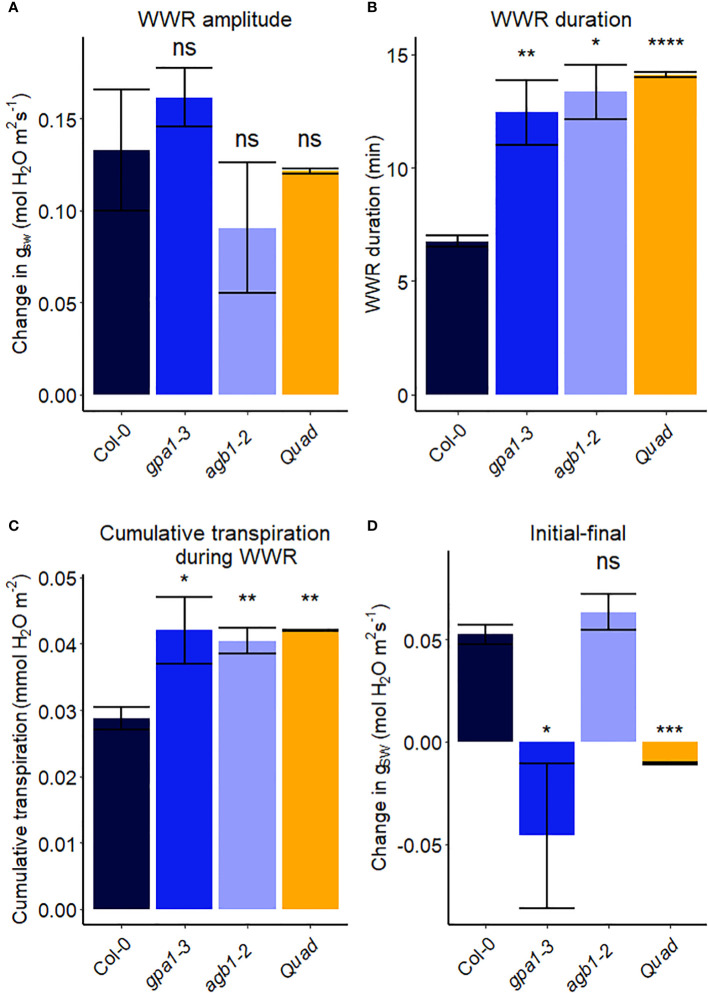
Stomatal VPD response characteristics in Arabidopsis WT (Col-0) and the mutants: *gpa1-3*, *agb1-2*, and Gα quadruple mutant *xlg1-1xlg2-1xlg3-4gpa1-3*. **(A)** The “wrong way response” (WWR) amplitude represents the differences in stomatal conductance at the time of the peak vs. before the VPD change. **(B)** The WWR duration represents the duration between the steady-state conductance before the VPD change and returning to the same baseline. **(C)** Cumulative water loss in transpiration during WWR. **(D)** Difference in steady-state g_sw_ values before and after the change in VPD. Asterisks indicate p-values and define significance levels as follows: * for p < 0.05; ** for p < 0.01; *** for p < 0.0001; ns, not significant.

**Figure 6:**
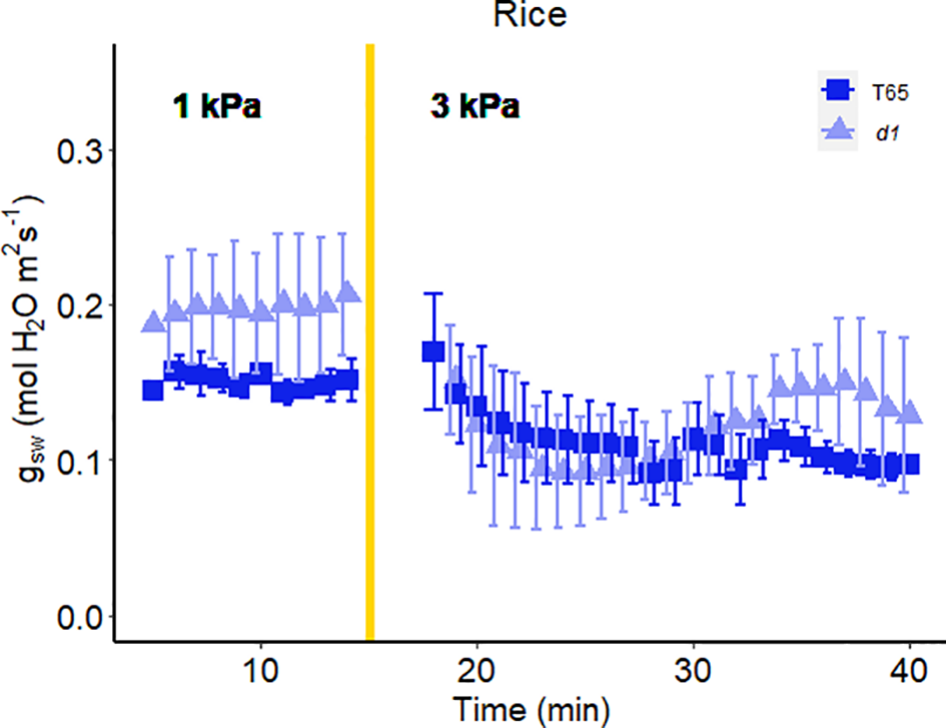
Effect of a sharp increase in vapor pressure difference (VPD) on stomatal conductance in rice T65 and *d1* mutants of the α-subunit of the rice heterotrimeric G protein, *RGA1*. The vertical line in the graph indicates the time at which the VPD was changed from 1 to 3 kPa. Error bars represent standard error of the mean (n=4).

In another experiment, we compared Arabidopsis Col-0 with the *gpa1-3* mutant (heterotrimeric G protein α subunit mutant), *agb1-2* (heterotrimeric G protein ß subunit mutant) and Gα quadruple mutant (Ding et al., 2008) (**Figure 4**). While the amplitude of the WWR did not differ among genotypes (**Figure 5A**), the duration of the WWR was longer in the *gpa1-3, agb1-2* and Gα quadruple mutants (**Figure 5B**), leading to increased cumulative water loss during the WWR in *agb1-2*, the Gα quadruple, and in *gpa1-3* (**Figure 5C**). A comparison of steady-state conductance before and after the step change in VPD showed VPD hyposensitivity of the *gpa1-3* and Gα quadruple mutants (**Figures 4A, C**, **5D**). The *agb1-2* mutant, on the other hand, ultimately showed a decrease in stomatal conductance under high VPD (**Figure 5D**), but with slower kinetics compared to Col-0 (**Figures 4B**, **5B, D**). These comparisons reveal an important role of heterotrimeric G proteins in the stomatal response to VPD. By contrast, when we tested the VPD response in the rice counterpart of the Arabidopsis *gpa1-3* mutant, *d1*, which is a protein null mutant of the Gα heterotrimeric G protein, *RGA1*, we did not observe similar trends as in the Arabidopsis *gpa1-3* mutant (**Figure 6**). Notably, the *d1* mutants exhibited elevated basal stomatal conductance (0.21 m^-2^ s^-1^ compared to 1.6 mol m^-2^ s^-1^). Furthermore, following the step change in VPD from 1 kPa to 3 kPa, the wild-type and *d1* plants displayed statistically similar new steady-state conductance levels after 30 min (~0.125 mol m^-2^ s^-1^). The *d1* plants also showed an oscillatory pattern of stomatal conductance, as seen by the increase in stomatal conductance between 30 min and 40 min.

### The role of receptor-like kinases in VPD sensitivity

**Figure 7:**
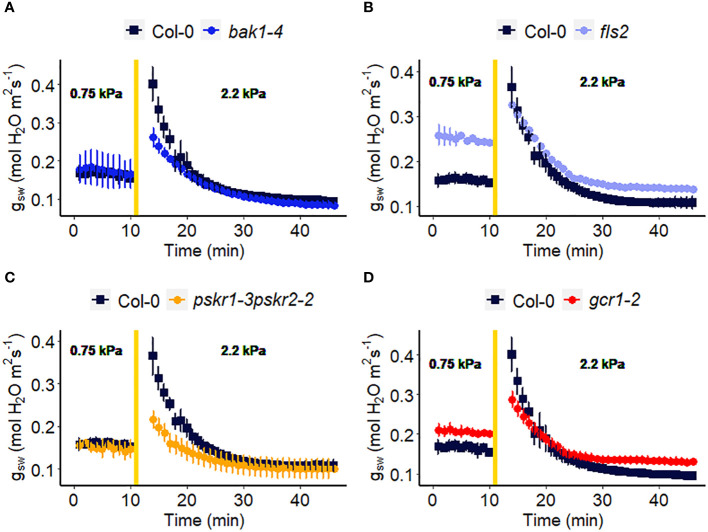
Effect of a sharp increase in vapor pressure deficit (VPD) on stomatal conductance in Arabidopsis WT (Col-0) and the mutants: **(A)** *bak1-4*, **(B)** *fls2*, **(C)** *pskr1-3pskr2-2*, and **(D)** *gcr1-2*. The vertical line in the graph indicates the time at which the VPD was changed from 0.75 to 2.2 kPa. Error bars represent standard error of the mean (n=3). For ease of comparison, the same Col-0 data are plotted in each of the 4 panels.

**Figure 8:**
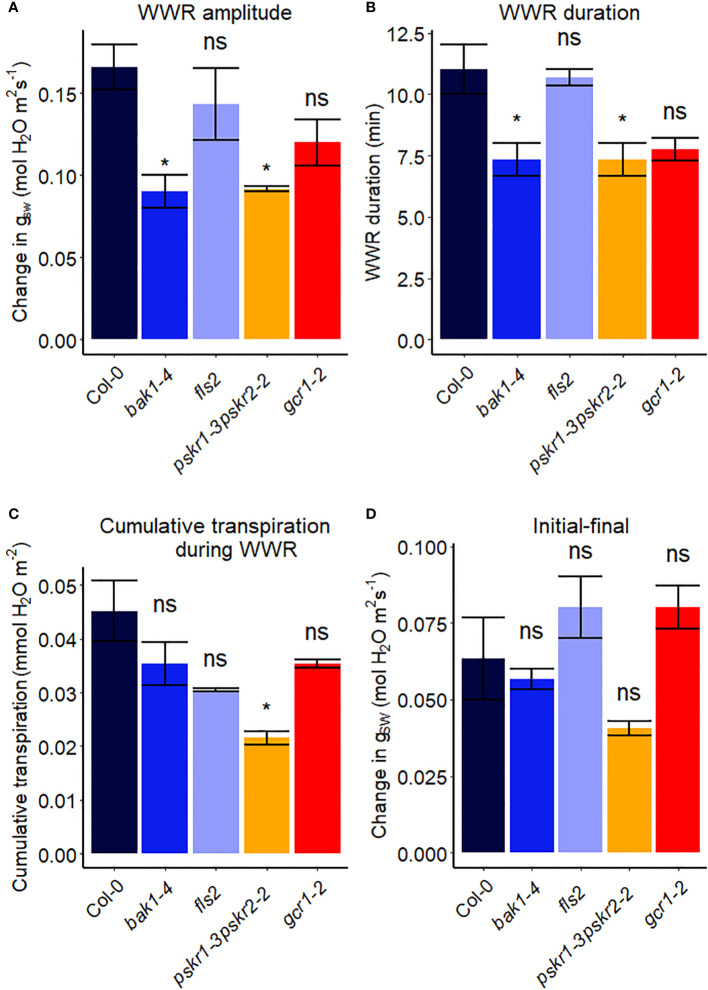
Stomatal VPD response characteristics in Arabidopsis WT (Col-0) and the mutants: mutants: *bak1-4*, *fls2*, *pskr1-3pskr2-2*, and *gcr1-2*. **(A)** The “wrong way response” (WWR) amplitude represents the differences in stomatal conductance at the time of the peak vs. before the VPD change. **(B)** The WWR duration represents the duration between the steady-state conductance before the VPD change and returning to the same baseline. **(C)** Cumulative water loss in transpiration during WWR. **(D)** Difference in steady-state g_sw_ values before and after the change in VPD. Asterisks indicate p-values and define significance levels as follows: * for p < 0.05; ns, not significant.

Receptor-like kinases (RLKs) have been implicated as receptors upstream of plant heterotrimeric G proteins (Ishida et al., 2014; Aranda-Sicilia et al., 2015; Chakravorty et al., 2018; Yu et al., 2018). In our final set of Arabidopsis experiments (**Figure 7**), we investigated the involvement of several Arabidopsis RLKs in the VPD response. The gas exchange experiment unveiled that the *pskr1-3pskr2-2 a*nd *bak1-4* mutants both exhibited significant lower WWR amplitude (**Figure 8A**), and shorter WWR time period (**Figure 8B**), which resulted in significantly lower WWR-mediated water loss in *pskr1-3pskr2-2* (**Figure 8C**). However, no significant differences in the change in steady-state conductance following a VPD step increase were observed among Col-0 and any of the RLK mutants (**Figures 7**, **8**).

### Stomatal response to VPD in different canola varieties

**Figure 9:**
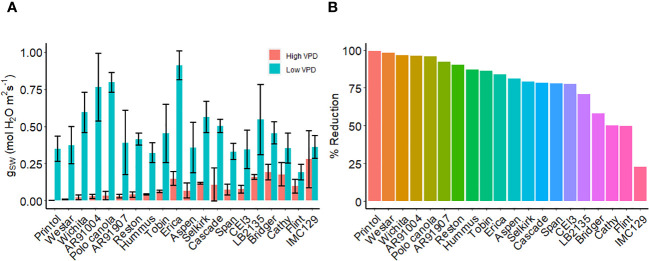
VPD response of canola cultivars. **(A)** Steady-state stomatal conductance, measured using the L-600 porometer, of multiple canola lines: AR91004, AR91907, Aspen, Bridger, Cascade, Cathy, CEI3, Erica, Flint, Hummus, IMC129, LB2135, Polo Canola, Printol, Reston, Selkirk, Span, Tobin, Webster, and Wichita. Conductance measurements were taken both before and two hours following exposure to high VPD condition. **(B)** The percent change in stomatal conductance after the exposure 2h exposure to high VPD. Error bars represent standard error of the mean (n = 4).

We examined various canola cultivars using porometry under high and low VPD conditions (**Figure 9A**). Notably, we identified a group of canola cultivars, including, IMC 129, Flint, Cathy, Bridger, and LB2315 which exhibited hyposensitivity to high VPD under these conditions (lines which showed reduction in g_sw_ of less than 75%) (**Figure 9B**). Conversely, we observed another set of canola lines, including Printol, Waster, Wichita, and AR91004, which showed strong sensitivity to VPD as stomatal conductance after 2 h of high VPD was nearly zero (**Figure 9A**).

## Discussion

The stomatal response to Vapor Pressure Difference (VPD) remains a complex and intriguing phenomenon. Guard cells have been proposed to sense various factors, including turgor loss, increased transpiration rates, or changes in humidity (Mott and Parkhurst, 1991).

Recent studies have suggested that the VPD response might actually be a dual one (McAdam and Brodribb, 2014; Merilo et al., 2018; Yaaran et al., 2019). One component appears to be mediated by the hormone ABA, triggered when leaf turgor decreases (McAdam and Brodribb, 2016). The other seems to represent the direct guard cell response to VPD, transpiration, and/or humidity, as observed in responses to VPD in isolated epidermal peels (Lösch, 1977; Shope et al., 2008). Several studies report that a guard cell response to VPD is observed in ABA-deficient and ABA-insensitive plants (Assmann et al., 2000), as well as in plants genetically engineered to have ABA-insensitivity restricted to the guard cells (Yaaran et al., 2019). Experiments that assay wilting presumably demonstrate a net effect of both responses, further influenced by hydraulic conductivity.

**Figure 10:**
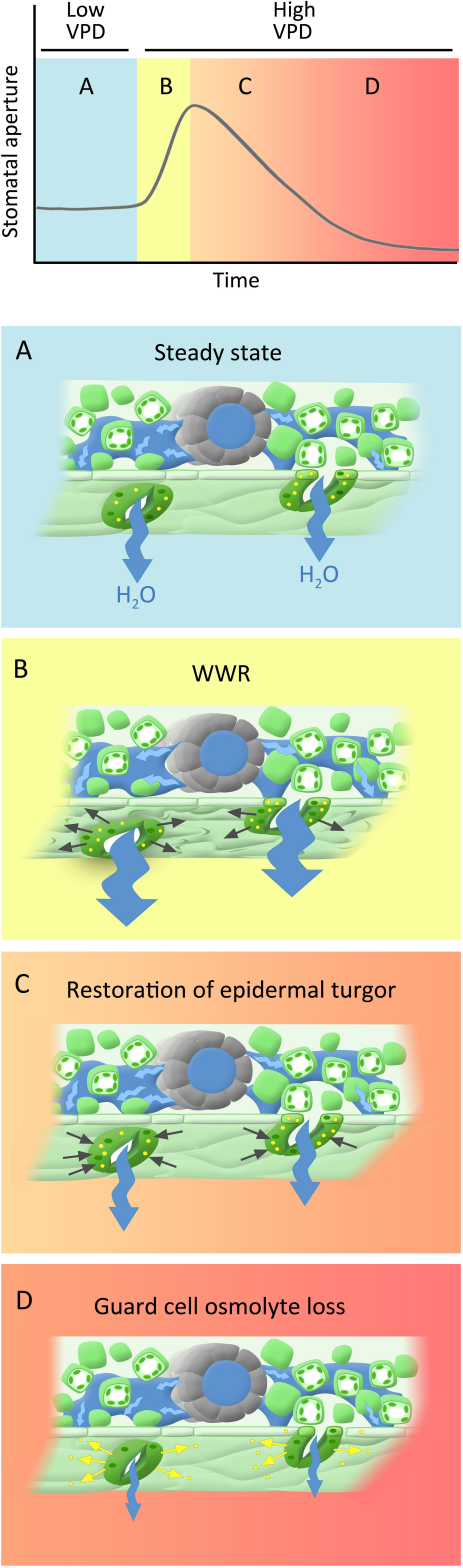
Phases of hypothesized dynamic stomatal response to VPD: **(A)** Steady state under low VPD conditions, characterized by high stomatal apertures, transpiration, and leaf turgor. **(B)** A rapid increase in VPD leads to increased transpiration, which creates a water deficit in the leaf tissues, leading to a decrease in turgor pressure within the epidermal cells, including those surrounding the stomata. This loss of turgor pressure passively causes stomatal opening (wrong way response; WWR). **(C)** Turgor restoration in pavement cells counters the initial aperture increase, a process significantly influenced by leaf hydraulic conductance (indicated by blue arrows). **(D)** The direct guard cell response to VPD, wherein the loss of osmolytes (yellow dots) from the guard cells triggers stomatal closure, serving as a protective mechanism that mitigates excessive water loss. As indicated by the color shading, phases C and D likely overlap temporally.

Here we show that part of the observed “wrong-way” response can be attributed to artifacts of the gas exchange measurement system (**Figure 1**). However, the remaining conductance changes comprise a genuine biological or biophysical response. We would like to propose a new, 3-component model to the guard cell VPD response (**Figure 10**). The first component consists of an abrupt increase in gs_w_ that is solely hydropassive, and that arises from water loss by neighboring epidermal cells that removes epidermal backpressure on guard cells, such that they gape open. In our experiments, this is reflected in the *amplitude* of the WWR. The second component involves restoration of turgor to the pavement cells of the epidermis, which opposes the initial g_sw_ increase; this component is strongly influenced by hydraulic conductance (Buckley et al., 2011), which in turn is influenced by ABA (Shatil-Cohen et al., 2011; Pantin et al., 2013a). The third component is the direct guard cell response to VPD, in which osmolyte loss from the guard cells promotes stomatal closure (Grantz and Zeiger, 1986; Buckley, 2005). This response is ABA independent or, stated more conservatively, does not required ABA sensing. The third component is quantified in our experiments as the initial g_sw_ minus the final g_sw_ i.e. the steady-state VPD response.

Support for the idea of the amplitude of the WWR as a separable component of the VPD response is provided by our data, in that we observe that some genotypes have a wild-type WWR amplitude but an impaired steady-state response to VPD (e.g. *ht1-1* and *ht1-2*), while other genotypes have a reduced WWR amplitude, but a wild-type steady-state response (e.g. *bak1-4* and *pskr1/pskr2*). In addition, according to this hypothesis, the second and the third components together dictate the *duration* of the WWR, and the associated extent of transpirational water loss. Differential relative contributions of these two components in different genotypes may influence the extent of observed ABA-dependence of the WWR, which may account for some of the debate in the literature regarding the ABA-dependence of guard cell VPD sensing. Finally, we note that the genotypic differences that we observe in the steady-state VPD response (*e.g.* in *gpa1* and the Gα quadruple vs. Col-0) are consistent with the proposition that turgor loss from guard cells is not simply hydropassive but involves mechanisms of VPD sensing and intracellular signaling, as also supported by the work of Hsu et al. (2021). Below, we further discuss how different Arabidopsis genes implicated in guard cell signaling influence these three components.

### HT1 kinase controls stomatal conductance in response to VPD

Our research endeavors to unravel the intricate mechanisms that govern stomatal responses to changes in VPD. We are particularly interested in exploring whether there exists a shared intracellular signaling pathway that modulates stomatal responses to both VPD and elevated CO_2_ level. Utilizing key CO_2_ hyposensitive (Wang et al., 2001; Tian et al., 2015; Kollist et al., 2018; Zhang et al., 2018) and abscisic acid (ABA) downstream component (Assmann et al., 2000; Merlot et al., 2001; Xie et al., 2006; Zhang et al., 2018) mutants of Arabidopsis, we aimed to shed light on the processes that catalyze active stomatal closure in response to high VPD conditions.

The *ht1-1* and *ht1-2* mutants, which harbor mutations in the *HIGH LEAF TEMPERATURE1* gene encoding a protein kinase expressed mainly in guard cells, showed reduced changes in g_sw_ in response to a step change in VPD as compared to Col-0 (**Figures 2**, **3**). This suggests that this intracellular signaling component is a shared element in stomatal response to VPD and elevated CO_2_. One hypothesis for the evolution of shared elements of the VPD and elevated CO_2_ response is that both factors play important roles in optimizing plant water use efficiency and carbon assimilation. Under conditions of high VPD, when the air is drier, stomata tend to close to reduce water loss and minimize desiccation stress (Mott and Parkhurst, 1991; Zait et al., 2017). This response is beneficial for conserving water resources. On the other hand, elevated CO_2_ concentrations promote more efficient photosynthesis and carbon assimilation in plants. To maximize the benefits of elevated CO_2_, stomata may partially close to limit the influx of CO_2_ and prevent excessive water loss through transpiration. Therefore, coordination between stomatal responses to VPD and elevated CO_2_ could have evolved, where changes in VPD may influence the response to CO_2_ and vice versa. Further research is needed to investigate the specific molecular mechanisms underlying this phenomenon and to assess other components in CO_2_ signaling for their potential role in the VPD response, such as MPK12 (Jakobson et al., 2016; Tõldsepp et al., 2018; Takahashi et al., 2022) and CBC1 (Hayashi et al., 2020; Ando et al., 2022). Surprisingly, given the central role of the SLAC1 ion channel in mediating anion loss in response to ABA and CO_2_, we did not observe an impaired VPD response in the *slac1* mutant. Lack of a difference in steady-state VPD responses of the *slac1* mutant was similarly evident in the data of Schroeder and colleagues (Hsu et al., 2021), although they did see slowed kinetics of the VPD-induced decrease in g_sw_ in *slac1*, which could arise from different growth conditions or initial leaf turgor status of their plants vs ours (Pantin et al., 2013b). Although HT1 is known to inhibit SLAC1 (Tian et al., 2015; Hõrak et al., 2016), it is possible that HT1 regulates the VPD response by modulating stomatal closure through a SLAC1-independent pathway. For example, other anion channels, such as QUAC1/ALMT2 might mediate solute loss during VPD-induced stomatal closure. Consistent with this idea, a *slac1 almt2* double mutant did show impaired steady-state stomatal closure following a step increase in VPD (Hsu et al., 2021). In addition, we observed that the H^+^ ATPase mutant, *aha1-6* has a reduced level of stomatal conductance under low VPD (**Figure 2F**), consistent with impaired light-induced stomatal opening (Yamauchi et al., 2016; Ando and Kinoshita, 2018), but shows little change in steady-state g_sw_ when VPD is increased (**Figure 2F**), thus also implicating H^+^ ATPases in the VPD response. We also showed that mutation of *RESISTANT TO HIGH CO_2_* (*RHC1*), which encodes a MATE-type transporter that links elevated CO_2_ levels to HT1 repression, has reduced sensitivity in stomatal response to high VPD (**Figures 2**, **3**). However, controversy surrounds the *rhc1* mutant response to elevated CO_2_, with conflicting findings in studies by Tian et al. (2015) and Tõldsepp et al. (2018), underscoring the need for additional investigations in this area.

A previous thermal imaging genetic screen identified an *ost1* EMS mutant as VPD hyposensitive based on its lower leaf temperature (Xie et al., 2006). Given that basal stomatal conductance was greater in this *ost1* mutant (Xie et al., 2006), it is not unexpected that it would be identified in such a screen. Xie et al. (2006) also reported slower decreases in stomatal conductance following an increase in VPD in these mutants relative to wild-type; final steady-state values in g_sw_ were not reported. Merilo et al. (2018) also observed a slowed VPD-induced g_sw_ decrease in an *ost1* mutant (*ost1-3*), and highlighted the possibility of both ABA-dependent and ABA-independent OST1 signaling in regulating VPD-induced stomatal closure. In contrast to these studies, our study found that the *ost1-3* mutant maintained sensitivity to high VPD similarly to wild-type plants in all components of the VPD-triggered g_sw_ timecourse. This result would be consistent with previous studies showing that ABA-deficient Arabidopsis, pea, and tomato (Merilo et al., 2018) as well as ABA-insensitive Arabidopsis plants (Assmann et al., 2000) or stomata (Yaaran et al., 2019) show a similar rates of high VPD-induced conductance decrease as wild-type plants. The contrasting findings on *OST1* can be attributed to several experimental differences. Firstly, our research employed a 2 cm^2^ vs the whole-plant chamber for gas exchange used by Merilo and colleagues. Moreover, the range of humidity changes imposed in our study was steeper, from 70% to 15% while in Merilo et al. (2018), air humidity decreased from approximately 70% to around 35%. This disparity underlines the potential complexity and context-dependency of plant responses to environmental factors and calls for further exploration of the mechanisms involved in stomatal regulation in response to VPD. Such context-dependency is seen, for example, in studies on poplar, where species with increased stomatal density and decreased guard cell size exhibit faster responses to VPD when grown in the greenhouse ((Durand et al., 2019); see also Caine et al., 2019)), but these correlations largely disappear when the same species are grown in the field (Durand et al., 2020). Growth conditions, specifically sun vs. shade growth environments also have been shown to affect how VPD influences stomatal conductance dynamics in response to sunflecks in the rainforest species, *Piper auritum* (Tinoco-Ojanguren and Pearcy, 1993). Illumination conditions during growth can also affect leaf size and thickness, which affect boundary layer thickness and thus the vapor pressure gradient experienced by leaves under natural conditions.

### The role of GPA1 in the plant’s response to VPD

We demonstrated that GPA1 plays a pivotal role in the VPD response (**Figures 4**, **5**). Notably, previous work placed emphasis on GPA1 involvement in both abscisic acid signaling (Wang et al., 2001) and carbon dioxide (CO_2_) response (Nilson and Assmann, 2010). The integration of ABA and CO_2_ signaling pathways, in which GPA1 plays a central part, may contribute to the guard cells’ ability to sense and respond to VPD changes. When activated, the G protein α subunit (Gα) binds GTP, causing the separation of the α subunit from the βγ subunit pair (Gβγ). Both Gα and Gβγ can interact with various downstream components of signaling pathways (Chakravorty and Assmann, 2019). Notably, K^+^ and Ca^2+^ channels are significant downstream effectors that are regulated by G proteins through both cytosolic signaling cascades and membrane-delimited pathways in plants (Wu and Assmann, 1994; Wang et al., 2001; Zhang et al., 2008; Zhang et al., 2011; Jeon et al., 2019). In the future, it will be important to test other transporters, ion channels, and aquaporins known to be involved in active stomatal closure, in the context of stomatal response to high VPD.

While Gα clearly influences the VPD response in Arabidopsis, its function in rice seems to differ. In rice, the *d1* mutant display reductions in stomatal conductance under high VPD conditions similar to WT, marked by oscillatory patterns in stomatal conductance (**Figure 6**). These observations may suggest a distinction in VPD sensing mechanisms between grasses and dicots, which could possibly be related to their differential guard cell morphology or cell wall composition. Guard cells in grasses such as rice display a characteristic dumbbell shaped morphology and are flanked by morphologically distinct subsidiary cells, which could influence the turgor balance between guard cells and the cells bordering them differently than in dicots. In addition, the cell wall has been shown to influence stomatal behavior (Rui et al., 2017; Chen et al., 2021) and both Arabidopsis GPA1 (Klopffleisch et al., 2011; McFarlane et al., 2021) and its rice ortholog RGA1 (Zait et al., 2021) have been implicated in modulating the cell wall, where VPD sensing may take place. Knockout of this canonical Gα subunit might have differential impacts on cell wall components in the two species, particularly given the well-known differences in cell wall composition between dicots and grasses (Vogel, 2008). Alternatively, different growth conditions for the two species may have contributed to the apparent disparity in the impact of Gα knockout, including greenhouse growth for rice and growth-chamber growth for Arabidopsis. In *Vicia faba*, for example, stomata of growth-chamber grown plants were more sensitive to CO_2_ than stomata of greenhouse-grown plants (Talbott et al., 1996), and difference in relative humidity in the two environments was identified as a key regulatory factor (Talbott et al., 2003).

In recent years, there has been growing evidence implicating receptor-like kinases (RLKs) in coupling with G protein signaling in plants (Chakravorty and Assmann, 2019). Genetic analysis has suggested potential interactions between RLKs and G protein subunits, and several studies have demonstrated physical interactions between specific RLKs and Gα or Gβγ subunits using techniques like targeted immunoprecipitation or bimolecular fluorescence complementation (Bommert et al., 2013; Ishida et al., 2014; Yu et al., 2018). In addition, phosphorylation of GPA1 by several RLKs has been demonstrated (Aranda-Sicilia et al., 2015). RLKs perceive extracellular peptide ligands. We speculate that under high VPD, the concentration of an apoplastic RLK ligand might effectively increase due to evaporation from the apoplast (Chawla et al., 2021). This increase in ligand concentration could serve as the signal for initiating stomatal closure in response to high VPD, coupling a physical phenomenon (VPD) with a chemical signal. Gas exchange measurements demonstrated that only *bak1-4* and *pskr1-3pskr2-2* mutants showed altered WWR patterns in response to high VPD, suggesting potential divergence in the underlying mechanisms of stomatal regulation among the *RLK* mutants. It is intriguing that across the 14 different Arabidopsis mutants assayed here for their VPD response, these two *rlk* mutants were the only genotypes to show a significant alteration in the amplitude of the WWR. In both cases, the WWR amplitude was decreased, which suggests the hypothesis that these mutations result in a decrease in the cuticular conductance, which in turn reduces the rate of passive loss of water through the cuticle in these mutants upon the step increase in VPD. The fact that none of the receptor mutants assayed in **Figures 7**, **8** phenocopied the VPD response of the *gpa1* mutant suggests that other receptors may function upstream of the G protein heterotrimer in the VPD response. While our research presents intriguing possibilities for understanding the coordination of responses to VPD and other stomatal signals through GPA1 interactions with RLKs, further research is needed to validate and elucidate specific molecular mechanisms.

### VPD response of canola cultivars

Canola is one of the crop species most closely related to the reference plant Arabidopsis, and it is reasonable to hypothesize that common VPD signaling mechanisms prevail in the two species. We observed variation in VPD sensitivity among different *Brassica napus* cultivars we assayed. Our results suggest that certain lines, such as Selkirk, span, and polo canola, exhibit hyposensitivity to high VPD. On the other hand, lines like Cascade, Tobin, and Hummus show heightened sensitivity to VPD. The large guard cells and easily removed epidermis of *Brassica napus* make it an ideal model crop for studying stomatal responses (Zhu et al., 2016a; Zhu et al., 2016b), including those to high VPD. Our observation of differential cultivar response to VPD emphasizes a genetic component to VPD sensitivity and suggests that GWAS approaches may be fruitful in identifying genes that encode components of VPD sensing and response. Unraveling the mechanisms through which stomata respond to VPD in *Brassica napus* could hold great potential for enhancing crop water-use efficiency and productivity, especially in the face of changing climates. Furthermore, as *Brassica napus* likely shares relevant genes with Arabidopsis, our findings may help to identify candidate genes or mutants that can be utilized for breeding or engineering more water-efficient crops for future agriculture.

## Data availability statement

The raw data supporting the conclusions of this article will be made available by the authors, without undue reservation.

## Author contributions

SA: Conceptualization, Formal analysis, Funding acquisition, Project administration, Supervision, Writing – original draft, Writing – review & editing. AJ: Investigation, Writing – review & editing. YZ: Conceptualization, Formal analysis, Funding acquisition, Investigation, Methodology, Supervision, Writing – original draft, Writing – review & editing.
