# Supplementary material for: Stomatal responses to VPD utilize guard cell intracellular signaling components
Source: Front Plant Sci. 2024 Feb 5;15:1351612. doi: 10.3389/fpls.2024.1351612 (PMC10875092; doi:10.3389/fpls.2024.1351612)
Supplement: Supplementary file 1 [file Table_1.pdf]

Supplementary material

Table S1: P values from t-test comparisons of the stomatal VPD response characteristics between Arabidopsis WT (Col-0) and the mutants.

Mutant	WWR amplitude	WWR duration	Cumulative transpiration during WWR	Initial-final gsw
ht1-1	0.51199	0.042172	0.49504	0.007716
ht1-2	0.123767	0.00077	0.242906	0.01491
slac1-3	0.397364	0.834672	0.276574	0.872525
ost1-3	0.829255	0.624719	0.266434	0.668646
ca1ca4	0.830032	0.3258	0.173307	0.433744
rhc1	0.139419	0.049416	0.938385	0.028626
aha1-6	0.433177	0.22511	0.535529	0.009502
gpa1-3	0.472926	0.007381	0.038238	0.032054
agb1-2	0.42812	0.027507	0.008045	0.355562
Quad	0.75553	0.000019	0.004044	0.000864
bak1-1	0.013748	0.045541	0.235638	0.670813
fls2	0.444269	0.776898	0.119107	0.378002
pskr1-1pskr2-1	0.03122	0.045541	0.046575	0.2288
gcr1-2	0.069979	0.063	0.218479	0.347297
